# Dominant Role of Habitat Transformation in Driving the Divergence of Health‐Risk Related Microbial Functional Genes in Karst Mountain Parks: A Metagenomic Study

**DOI:** 10.1002/ece3.74112

**Published:** 2026-07-29

**Authors:** Weize Wang, Chunhua Cen, Jingyi Yang

**Affiliations:** ^1^ College of Forestry, Institute for Forest Resources and Environment of Guizhou, Guizhou Key Laboratory of Forest Cultivation in Plateau Mountain, Guizhou University Guiyang China; ^2^ Guizhou Guiyang Urban Ecosystem Observation and Research Station, National Forestry and Grassland Administration Guiyang China

**Keywords:** habitat transformation, health‐related functional genes, karst mountainous park, plant–soil‐microbe interactions, soil health risk, urban soil microbiome

## Abstract

The transformation of natural forests into urban parks has had a profound impact on subterranean ecosystems. Nevertheless, the underlying mechanisms by which this land use change affects human health through alterations in soil microbial functional genes remain to be elucidated. Focusing on a karst mountain park in Guiyang, China, we used metagenomic sequencing to compare the abundance and composition of antibiotic resistance genes (ARGs), pathogen‐host interaction genes (PHIs), and virulence factor genes (VFs) between remnant forests and artificial green spaces, and examined how plant diversity and soil chemometrics drove their variation. Habitat type emerged as the strongest driver of gene composition. PHIs and VFs were more abundant in remnant forests and positively correlated with native plant diversity, while ARGs were enriched in artificial green spaces. All three gene categories showed positive correlations with soil nitrogen content in artificial green spaces. Remnant forests harbored microbial functions linked to complex plant–microbe interactions, whereas intensive management in artificial green spaces selects for antibiotic resistance and nutrient‐adaptive genes. These findings reveal distinct health risks across habitats, suggesting that differentiated park management strategies are needed to mitigate public health risks while maintaining ecological sustainability.

## Graphical abstract

This manuscript elucidates the impact of transforming karst mountain forests into urban parks on health‐risk related microbial functional genes and its implications for mitigating potential public health exposures. Distinctively, the study advances prior research by evaluating the variation in health‐related functional genes within parks of varying ages and contrasting habitat types (remnant forests versus artificial green spaces). Furthermore, it identifies the critical factors shaping the functional gene assemblage and compare these patterns between the two habitat types.

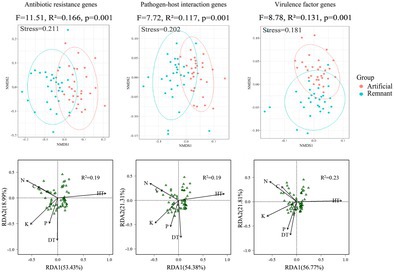

## 1 Introduction

Karst landscapes, formed by carbonate bedrock dissolution, hold high ecological value due to their biodiversity and sensitivity (Zhang et al. 2010; Liu et al. 2018). Guiyang, characterized by extensive remnant limestone forests embedded within the urban matrix, is a typical karst mountainous city situated in one of the world's largest and most ecologically fragile karst regions (Du et al. 2017; Hu et al. 2023; Wu et al. 2022; Liao et al. 2022; Yang et al. 2022). Rapid urbanization has fragmented these forests; to balance development and green space demand, Guiyang converted some remnants into urban mountain parks (Wang and Yang 2022; Luo et al. 2022). This habitat shift alters soil microbial taxonomy and α‐diversity, reducing bacterial/archaeal richness while increasing evenness, shifting fungal communities, and simplifying symbiotic networks—indicating declined ecosystem stability (Cen et al. 2025). Antibiotic resistance genes (ARGs) from disturbed soils can transfer to human pathogens via food chains, aerosols, or direct contact, posing public health threats (Zhu et al. 2019). In contrast, microbial diversity in remnant forests supports immune training and prevents non‐communicable diseases (Rook 2013; Wall et al. 2015). Therefore, investigating habitat‐transformation‐driven changes in health‐risk‐related microbial functional genes is critical for assessing urban green space impacts on public health.

Recent studies have established key ecological patterns arising from such land‐use conversion. Above ground, the transformation leads to homogenized woody plant composition in parks, whereas remnant forests maintain higher native species diversity and regeneration capacity, and artificial green spaces are dominated by ornamental species (Wang, Gao, et al. 2024). Below ground, habitat conversion significantly alters soil chemistry and reshapes the relationships between soil properties and plant diversity across the two habitats (Jian et al. 2025). It also restructures soil microbial communities, affecting functional gene composition and reducing the complexity of co‐occurrence networks (Cen et al. 2025). While microbial communities in artificial green spaces display enhanced metabolic potential, they show reduced taxonomic diversity (Yang et al. 2024; Cen et al. 2025).

However, prior work has focused mainly on microbial roles in core biogeochemical cycles (e.g., carbon and nitrogen), leaving largely unexplored those functional genes with direct implications for public health risks. Urban green spaces are essential elements of the urban ecosystem, offering residents vital recreational areas while helping to mitigate environmental stressors and sustain microbial diversity (Chen et al. 2023; Elgizawy 2014; Yang et al. 2021). Microbial diversity plays a central role in nutrient cycling, organic matter decomposition, and the regulation of ecological immune functions, thereby underpinning ecosystem health and stability (Xu et al. 2015; Rook 2013). Soils in urban parks form a dynamic and complex microbial reservoir, with their community structure and functional potential being profoundly shaped by anthropogenic activities (Setälä et al. 2016).

Of particular concern are specific functional genes carried by soil microorganisms—such as ARGs, pathogen–host interaction genes (PHIs), and virulence factor genes (VFs)—which are directly linked to public health. These genes may pose risks to park visitors through direct contact (e.g., skin exposure) or indirect pathways (e.g., aerosol transmission) (Tiedje et al. 2019; Echeverría‐Palencia et al. 2017). Thus, the occurrence, distribution, and drivers of these health‐relevant genes in urban green spaces represent a critical emerging dimension in assessing urban ecosystem health, serving as a key interface between ecological processes and potential public health outcomes.

In urban settings, particularly within highly frequented parks and green spaces, understanding the distribution patterns of health‐related functional genes is of practical urgency. Microbial community assembly and co‐occurrence networks differ markedly between habitat types (Wang, Feng, et al. 2024). The complex and stable microbial networks in remnant forests may strongly resist the colonization of foreign ARGs and PHIs (Zhi et al. 2024). In contrast, artificial green spaces often harbor higher abundances of ARGs, likely due to frequent anthropogenic disturbance and inputs such as pet waste (Beroigui et al. 2020). Moreover, the application of fertilizers can selectively promote certain microbial taxa by altering nutrient availability (Bei et al. 2018).

As parks age, soil microbial communities tend to stabilize, forming niches that facilitate pathogen colonization. Over time, microorganisms carrying ARGs and PHIs may become dominant, further promoting the spread of these genes through horizontal gene transfer (Shen et al. 2024; Zhao et al. 2025). Together, these factors profoundly shape the transmission and spatial distribution of the three health‐relevant functional gene categories.

In this study, we aimed to address the following scientific questions: What are the differences in the composition and abundance of antibiotic resistance, pathogen interaction, and virulence‐related functional genes in soils between remnant forests and artificial green spaces within karst mountainous urban parks? What are the primary drivers of these differences? We established three specific research objectives: First, to compare the compositional and abundance differences of the three health‐relevant functional gene categories both among parks and between the two habitat types. Second, to quantify and distinguish the relative contributions of habitat type, park age, soil chemical properties, and plant diversity to the variation in the composition of these three functional gene categories. Third, to elucidate the association patterns between the abundance of key functional genes and environmental factors, and to compare these patterns between the two habitat types. We hypothesized that habitat type is the predominant factor driving the divergence in the composition of the three health‐relevant functional gene categories. Specifically, due to long‐term nutrient inputs and management disturbances, soils in artificial green spaces would exhibit significantly higher abundances of ARGs compared to remnant forests. Conversely, remnant forests, characterized by more complex plant‐microbe interaction networks, were predicted to harbor higher abundances of PHIs and VFs. In addition, we predict that the abundance of all three types of functional genes in artificial green spaces is significantly positively correlated with soil nitrogen content, indicating that anthropogenic nutrient enrichment is a key factor driving the accumulation of health‐risk genes, while in remnant forests, functional gene abundances were positively correlated with plant diversity.

## 2 Materials and Methods

### 2.1 Study Area

**FIGURE 1:**
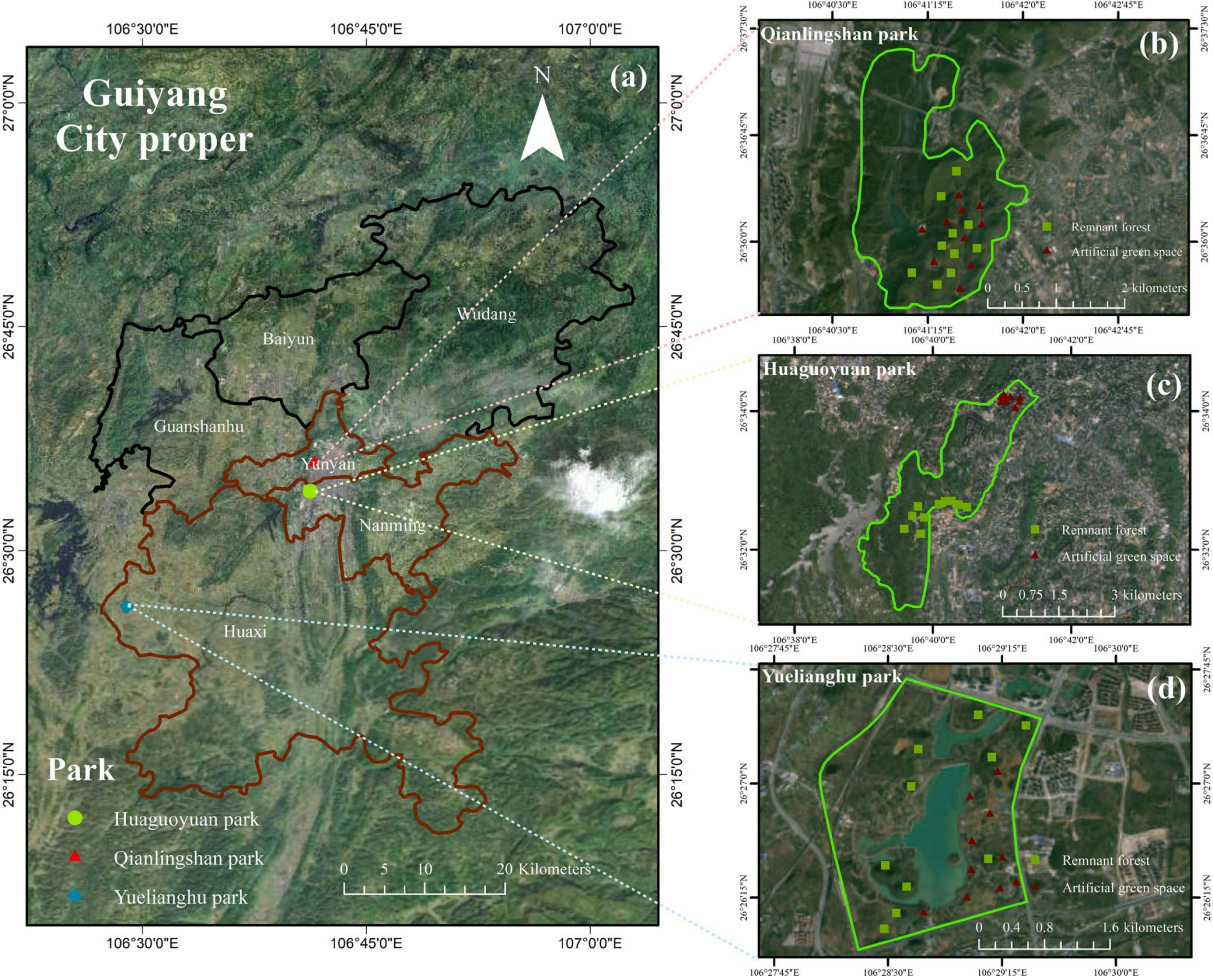
The study area (a), spatial location of sample plots in (b) Qianlingshan, (c) Huaguoyuan and (d) Yuelianghu park.

This study was implemented in Guiyang City, Guizhou Province (Figure 1a), a representative karst mountainous city in southwestern China. Located on the Yunnan‐Guizhou Plateau, the city has an average elevation of approximately 1100 m and features a subtropical humid temperate climate, with a mean annual temperature of 15.3°C and annual precipitation of 1046 mm. Due to intense karst geomorphological development and historical urbanization, numerous patchily distributed remnant mountain forests have been preserved within the urban matrix. To meet public recreational needs, many of these areas have been transformed into urban mountain parks that integrate natural woodland with artificial landscapes. Based on their establishment dates, three parks were selected as study sites: Qianlingshan Park (established 1957; Figure 1b), Huaguoyuan Park (established 2010; Figure 1c), and Yuelianghu Park (established 2020; Figure 1d). Within each park, two contrasting habitat types were clearly identified: (1) remnant forests, which remain largely in a natural state with minimal anthropogenic disturbance, and (2) artificial green spaces, which are designed landscapes dominated by ornamental plants and receive regular maintenance.

### 2.2 Field Survey and Data Collection

This study was conducted from June to August 2023 and included a comprehensive survey of aboveground vegetation and subsurface soil sampling for microbial analysis. The survey encompassed three parks in Guiyang City: Qianlingshan Park, Huaguoyuan Park, and Yuelianghu Park. Within each park, systematic sampling areas were established across two contrasting habitats based on their origin and management: remnant forests and artificial green spaces. Within each habitat, 10 systematic quadrats, each measuring 20 m × 20 m, were randomly established, resulting in a total of 60 quadrats (Figure 1b–d). This design established a research framework incorporating the two factors of park and habitat type, yielding six distinct treatment groups. In all quadrats, a complete census was performed for all woody plants with a diameter at breast height greater than 1 cm. Species identity and abundance were recorded for each woody plant species. The Shannon–Wiener index was subsequently calculated to quantify plant community diversity.

Soil sampling was conducted concurrently with the vegetation survey. Within each quadrat, a composite topsoil sample (0–10 cm depth) was obtained using a five‐point mixed sampling method. This layer of soil has a high concentration of health‐related microbial functional genes (ARGs, PHIs, VFs) due to human input, and the microbial activity is the highest. It is the main exposure interface for direct contact between the park soil and visitors (Delgado‐Baquerizo et al. 2022; Shang et al. 2025). Subsamples from the five points were thoroughly homogenized to form one representative composite sample per quadrat, yielding a total of 60 composite soil samples. All samples were transported to the laboratory within 12 h of collection for processing. One portion of fresh soil was immediately stored at −80°C for subsequent total soil DNA extraction and metagenomic sequencing analysis. The remaining soil was air‐dried naturally, ground, and sieved through a 0.15 mm mesh for the determination of key chemical properties. This included soil organic carbon (determined by the potassium dichromate external heating method), total nitrogen (Kjeldahl method), total phosphorus (molybdenum blue colorimetry), and total potassium (NaOH fusion‐flame photometry), all following the analytical methods described in Analytical Methods for Soil Agrochemistry (Lu 1999).

### 2.3 DNA Extraction, Library Construction, and Metagenomics Sequencing

To enable high‐throughput profiling of the soil microbiome, a standardized molecular workflow was implemented. Total genomic DNA was efficiently extracted from each 0.25 g fresh soil sample using an optimized protocol with the Tiangen DP705 Kit on the TGuide S96 Automated Nucleic Acid Extraction and Purification System. Rigorous quality control steps followed: DNA concentration was accurately quantified with a Qubit 3.0 Fluorometer using the dsDNA HS Assay Kit, requiring a minimum concentration of 5 ng/μL and a total yield of ≥ 0.5 μg. Integrity was assessed via 1% agarose gel electrophoresis, where a dominant, clear band above 5 kb without significant degradation or impurity was mandatory. Qualified DNA proceeded to library preparation using the VAHTs Universal Plus DNA Library Prep Kit for Illumina, adhering strictly to the manufacturer's guidelines. The resulting libraries were first evaluated for fragment size distribution on a Qsep‐400 Bioanalyzer, with the main peak required to fall within 430–530 bp. Library concentration was re‐quantified using the Qubit 3.0, demanding ≥ 1 ng/μL and a normal peak shape free of non‐specific products. Finally, all qualified libraries were subjected to high‐throughput sequencing on an Illumina NovaSeq 6000 platform employing a paired‐end 150 bp (PE150) strategy to generate high‐quality metagenomic raw data.

### 2.4 Metagenomic Quality Control and Assembly

Metagenomic quality control and assembly were conducted through a streamlined bioinformatic pipeline. Following sequencing, raw reads underwent stringent quality control and adapter trimming using Trimmomatic (v0.33) to ensure data integrity. Subsequently, a de novo co‐assembly of all high‐quality reads was performed with MEGAHIT (v1.1.2), a tool optimized for metagenomic datasets, which discarded contigs shorter than 300 bp. The quality of the final assembly was comprehensively evaluated using QUAST (v2.3) to assess key contiguity and completeness metrics.

### 2.5 Functional Annotation

To gain deep insights into the metabolic potential and ecological functions of the soil microbial communities, a systematic functional annotation was performed on the assembled non‐redundant gene catalog. Initially, protein sequences were aligned against the NCBI NR database using DIAMOND software (v0.8.35) with stringent parameters (*e*‐value ≤ 1e‐5) to obtain taxonomic information and homologous annotations. Building upon this, a multi‐database annotation strategy was employed for a comprehensive functional landscape: metabolic and signaling pathways were annotated via the KEGG database; orthologous protein families were classified using eggNOG; genes encoding carbohydrate‐active enzymes were identified with the CAZy database. Furthermore, targeting ecologically and health‐relevant functions central to this study, specific databases were utilized: ARGs were identified using the CARD database, VFs were annotated via the VFDB, and genes associated with pathogen–host interactions were analyzed using the PHI database. Each sample was sequenced at 6 G with coverage > 0.99, and although no technical replicates were performed, each habitat type included 10 independent biological replicates (composite soil cores), which has been widely accepted in soil metagenomic studies as sufficient to capture reliable ecological patterns (Table S1).

### 2.6 Data Analysis

**FIGURE 2:**
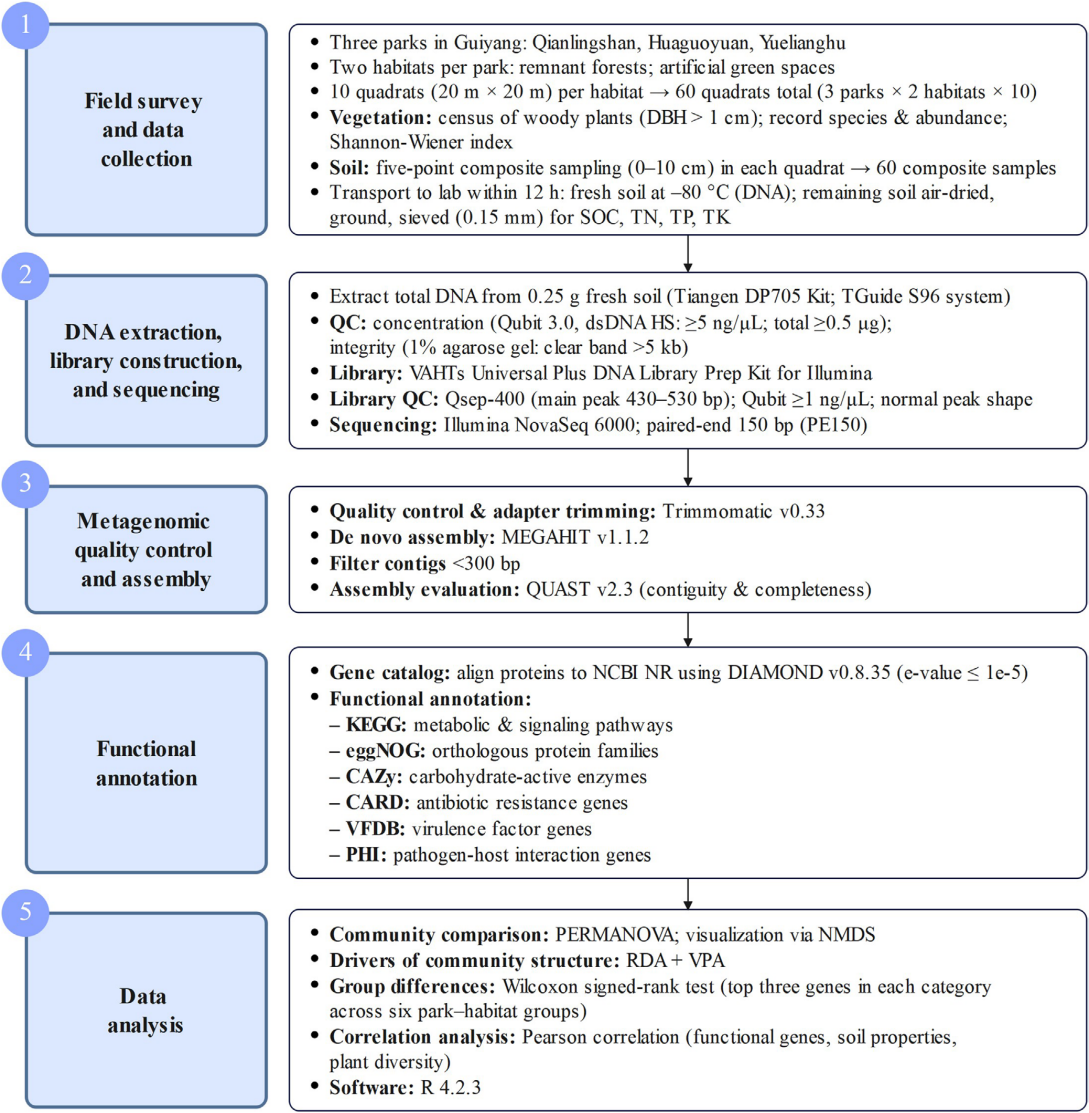
Workflow diagram.

The comparative analysis of ARG, PHI, and VF compositions between the two habitat types across three parks was performed using permutational multivariate analysis of variance (PERMANOVA) to test for significant compositional dissimilarities, the results of which were visualized using non‐metric multidimensional scaling (NMDS). To identify the key drivers shaping the community structure of these three gene categories, redundancy analysis (RDA) combined with variance partitioning analysis (VPA) was conducted. Differences in the relative abundance of the top three most abundant genes within each category among the six park‐habitat groups were assessed using the Wilcoxon signed‐rank test. Furthermore, Pearson correlation analysis was employed to examine the relationships between the abundance of these functional genes, soil chemical properties, and plant diversity. All statistical analyses were performed in R software (version 4.2.3) (R Core Team^2019^). The workflow of this study is summarized in Figure 2.

## 3 Results

### 3.1 Compositional Dissimilarities of Functional Genes in Three Parks Across Two Habitats

**FIGURE 3:**
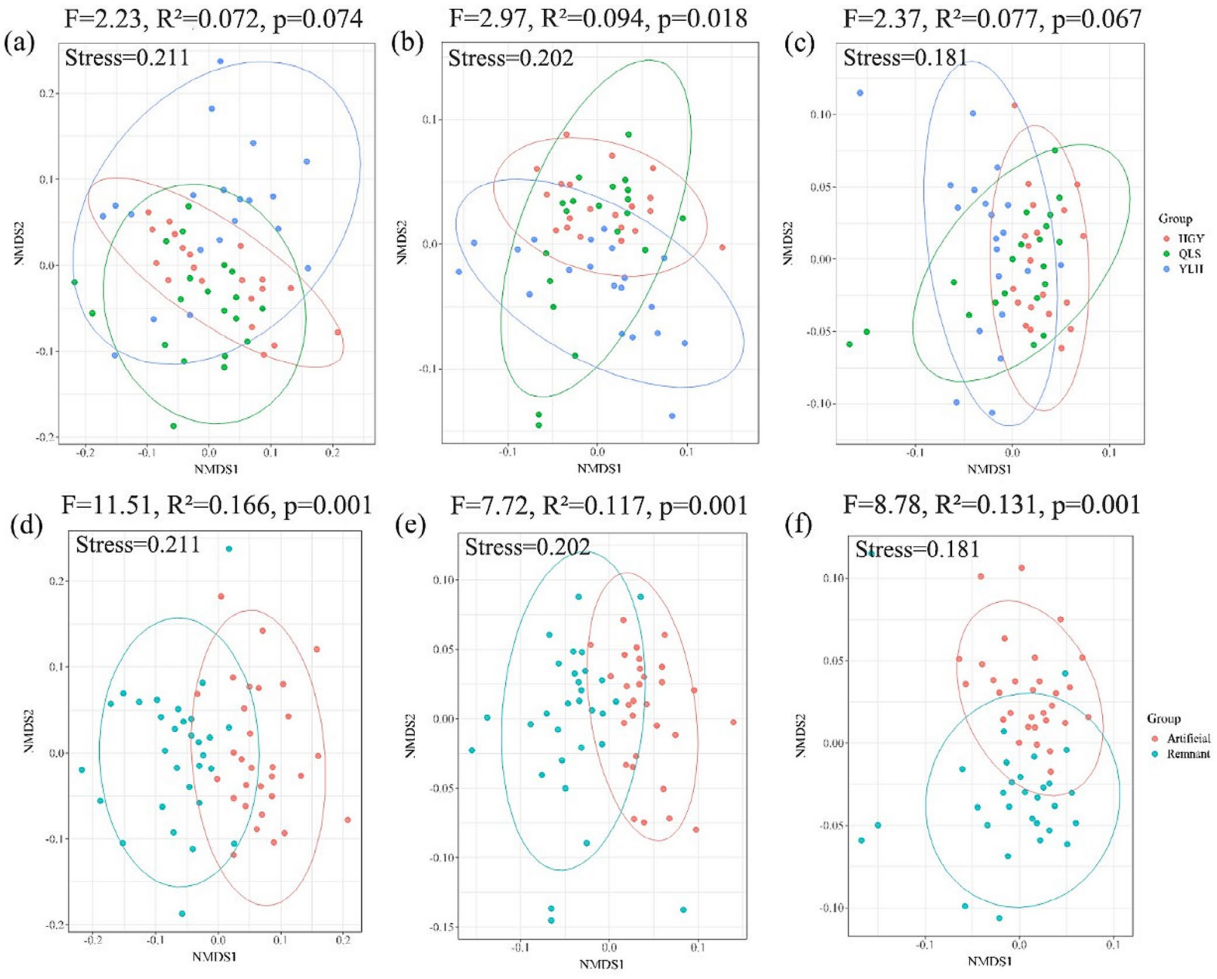
Compositional differences of three types of functional genes. (a, d) Antibiotic resistance genes; (b, e) pathogen–host interaction genes; (c, f) virulence factor genes. HA denotes artificial green spaces in Huaguoyuan Park; HGY, QLS, and YLH represent Huaguoyuan, Qianlingshan, and Yuelianghu parks, respectively.

The results showed that there was no significant difference in the composition of ARGs and VFs among the three parks (Figure 3a,c, *p* > 0.05). However, when park identity was disregarded and samples were classified into the two habitat types, the compositional differences in the three functional gene categories between the two habitat types became significant (Figure 3d–f, *p* < 0.05).

### 3.2 Factors Influencing the Composition of the Functional Genes

**FIGURE 4:**
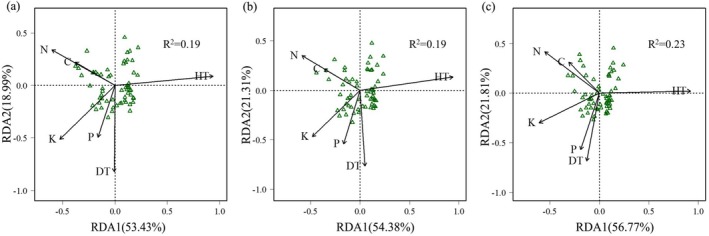
RDA results showing drivers of functional gene composition: (a) antibiotic resistance genes, (b) pathogen–host interaction genes, (c) virulence factor genes. C, soil organic carbon; DT, park development time; HT, habitat type; K, soil potassium; P, soil total phosphorus; N, soil total nitrogen.

Redundancy analysis and variation partitioning revealed that habitat type was the most important factor shaping the composition of the three functional gene categories, explaining 13%–15% of the variation (Figure 4, Table 1). Among the soil chemical properties, total potassium and total nitrogen contents also exerted substantial influence on the gene composition, accounting for 6%–8% of the explained variation (Table 1).

**TABLE 1:** Variance partitioning of factors influencing the composition of three functional gene categories.

		HT	DT	K	C	P	N
ARGs	*R* ^2^	**0.13**	0.04	**0.06**	0.03	0.03	**0.07**
	*F* value	8.61	2.66	3.98	1.91	1.83	4.09
	*p* value	0.001	0.002	0.001	0.019	0.019	0.001
PHIs	*R* ^2^	**0.13**	0.05	**0.06**	0.03	0.03	**0.07**
	*F* value	8.98	2.78	3.71	1.83	1.97	4.09
	*p* value	0.001	0.004	0.002	0.042	0.022	0.001
VFs	*R* ^2^	**0.15**	0.05	**0.08**	0.03	0.04	**0.07**
	*F* value	10.27	3.02	5.12	1.79	2.27	4.37
	*p* value	0.001	0.003	0.001	0.083	0.022	0.001

*Note:* The bolded number represents the R^2^ values of the main factors influencing the composition of three types of functional genes.

### 3.3 Differences in the Abundance of Three Functional Gene Types Across Different Habitats

**FIGURE 5:**
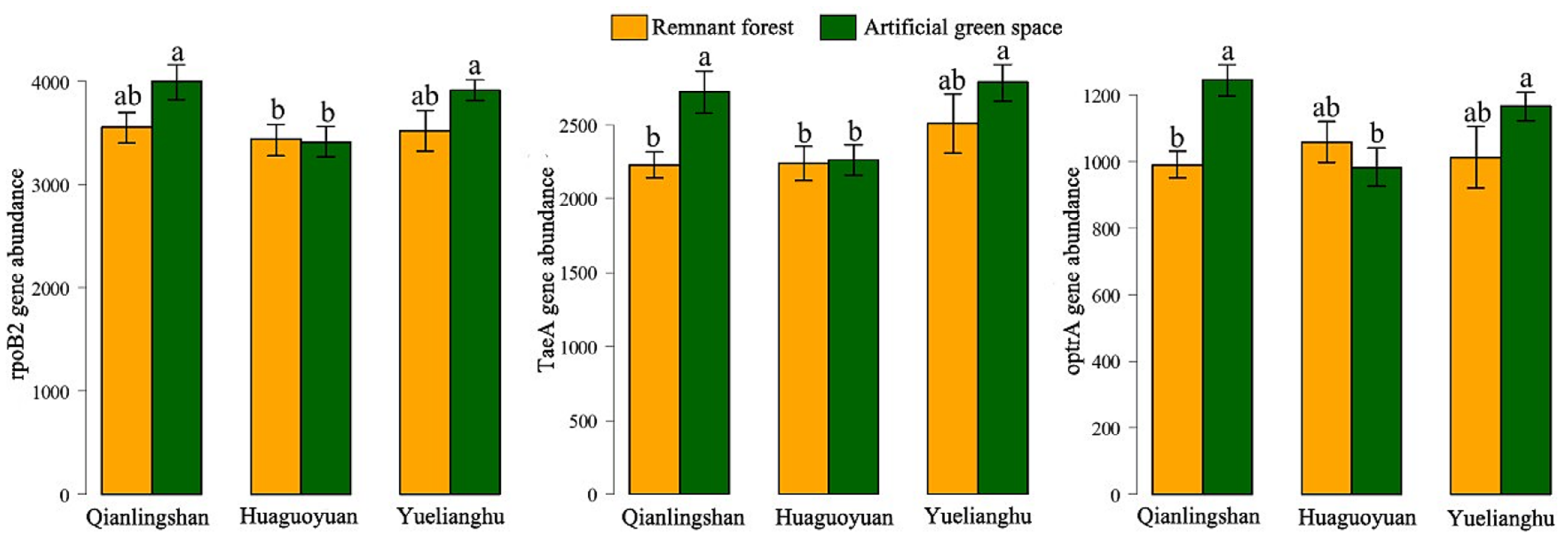
Abundance comparison of the top three most abundant ARGs across two habitat types in three parks. Different letters indicate significant differences (*p* < 0.05). rpoB2: rpoB2conferring resistance to peptide and rifamycin antibiotics; TaeA: taeAconferring resistance to pleuromutilin antibiotics; optrA: optrAconferring resistance to oxazolidinone and phenicol antibiotics.

**FIGURE 6:**
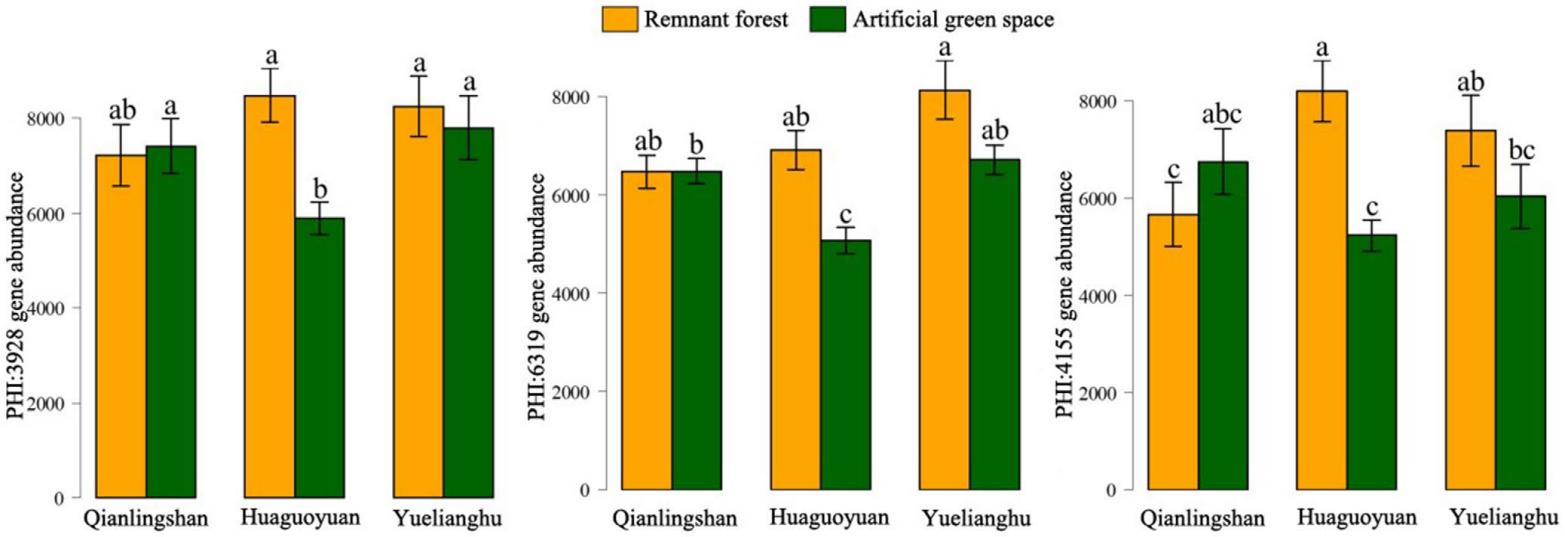
Abundance comparison of the top three most abundant PHIs across two habitat types in three parks. PHI:3928: MacBfrom Salmonella enterica; PHI:6319: PotAfrom Streptococcus pneumoniae; PHI:4155: Stkfrom Streptococcus suis.

**FIGURE 7:**
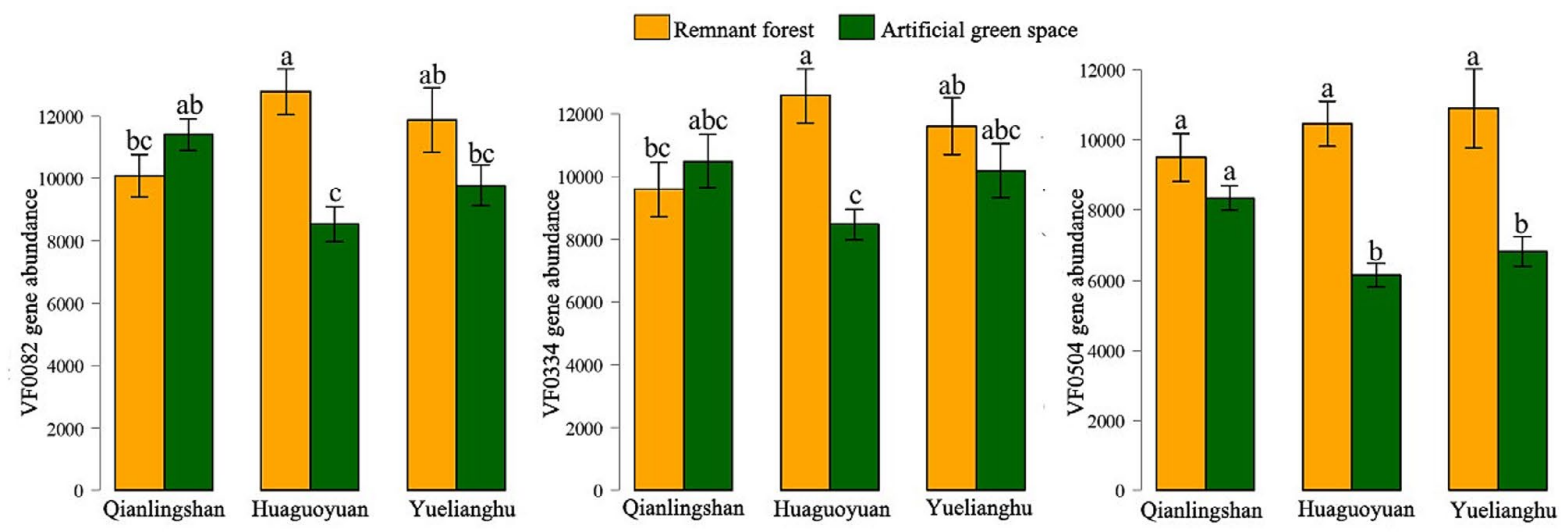
Abundance comparison of the top three most abundant VFs across two habitat types in three parks. VF0082: pilSencoding two‐component sensor PilS; VF0334: ppkAencoding serine/threonine protein kinase PpkA; VF0504: adeGencoding cation/multidrug efflux pump.

Multiple comparison results revealed that the three most abundant ARGs (optrA conferring resistance to oxazolidinone and phenicol antibiotics, taeA conferring resistance to pleuromutilin antibiotics, and rpoB2 conferring resistance to peptide and rifamycin antibiotics) exhibited higher abundance in artificial green spaces, with significant differences observed between the two habitat types in Qianlingshan Park (Figure 5). In contrast, PHIs (Stkfrom *Streptococcus suis* , PotAfrom *Streptococcus pneumoniae* , MacBfrom *Salmonella enterica* ) and VFs (adeGencoding cation/multidrug efflux pump, ppkAencoding serine/threonine protein kinase PpkA, pilSencoding two‐component sensor PilS) were more abundant in remnant forests, showing significant differences between habitats in Huaguoyuan Park (Figures 6 and 7).

### 3.4 Functional Gene Abundance in Relation to Soil Properties and Plant Diversity

**FIGURE 8:**
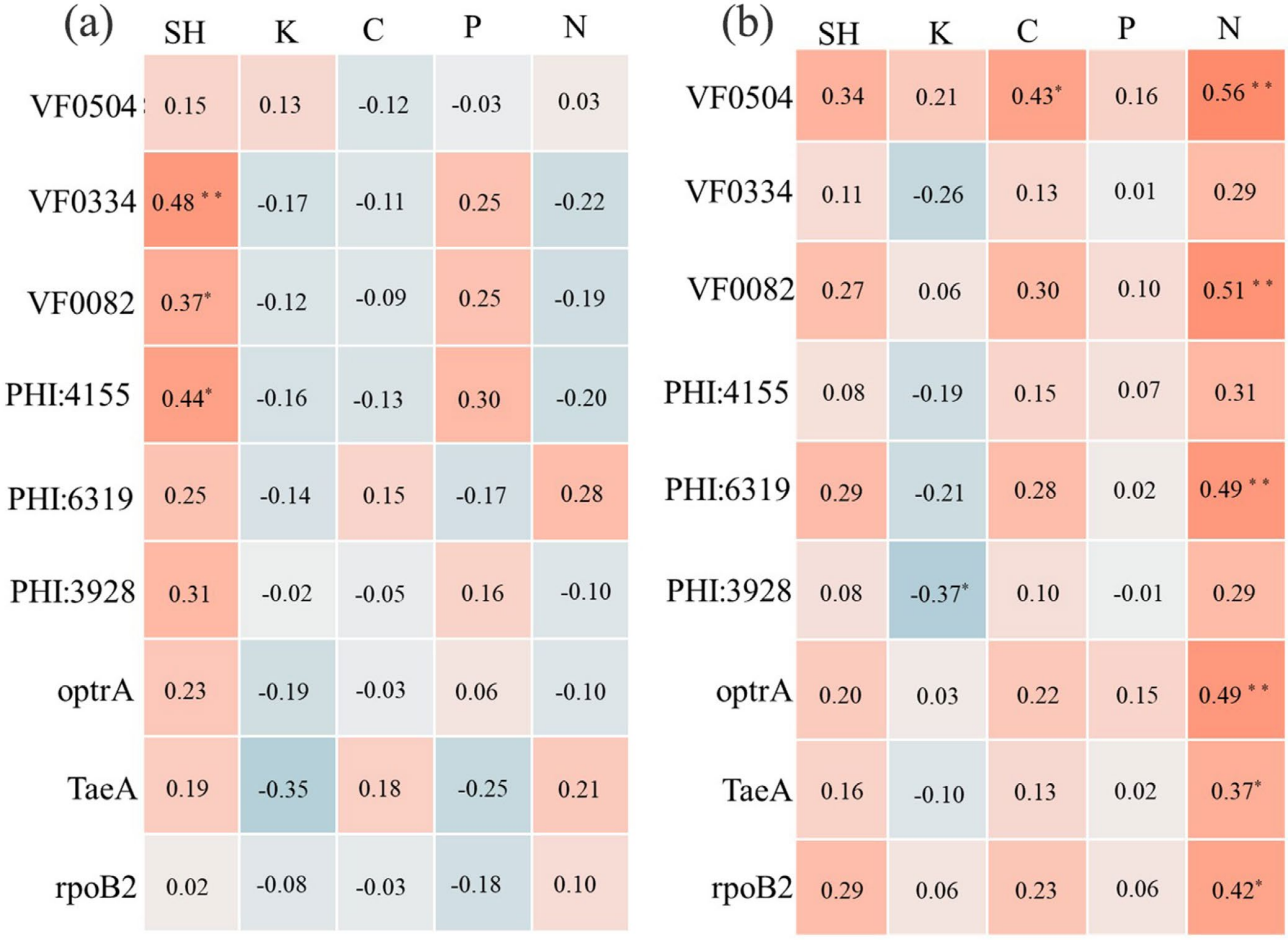
Pearson correlation between abundances of the top three genes in each category and plant diversity/soil chemistry in (a) remnant forests and (b) artificial green spaces. C, soil organic carbon; K, soil potassium; N, soil total nitrogen; P, soil total phosphorus; SH, Shannon diversity index of woody plants. **p < 0.01; *p < 0.05.

Pearson correlation analysis revealed that in remnant forests, the abundance of PHIs (Stkfrom *Streptococcus suis* ) and VFs (ppkAencoding serine/threonine protein kinase PpkA, pilSencoding two‐component sensor PilS) was positively correlated with plant species diversity (Figure 8a). Conversely, in artificial green spaces, the abundance of genes across all three functional categories showed a positive correlation with soil nitrogen content (Figure 8b), including the aforementioned pathogen–host interaction and VFs (adeGencoding cation/multidrug efflux pump, pilSencoding two‐component sensor PilS, PotAfrom Streptococcus pneumoniae), as well as ARGs (optrA conferring resistance to oxazolidinone and phenicol antibiotics, taeA conferring resistance to pleuromutilin antibiotics, rpoB2 conferring resistance to peptide and rifamycin antibiotics).

## 4 Discussion

The results of this study indicate that the composition of microbial functional genes related to health risks in karst mountainous urban parks is not determined by individual characteristics of the parks, but is fundamentally regulated by the type of habitat. This finding aligns with established knowledge regarding the ecological characteristics of these habitats. Remnant forests typically feature complex vegetation structures, high tree species richness dominated by native plants, and minimal human intervention (Wang, Gao, et al. 2024). Such diverse plant communities and stable soil environments are known to support a greater abundance of microbial functional genes associated with natural biotic interactions and ecological adaptation, such as those related to pathogenicity and host interactions (Chen et al. 2019; Dhungana et al. 2023). Conversely, artificial green spaces are characterized by simplified vegetation structures, a predominance of ornamental species, and frequent human management (e.g., fertilization, irrigation, pruning) (Wang, Gao, et al. 2024). These anthropogenic disturbances often lead to homogenized soil microbial communities and an enrichment of functional genes linked to metabolic adaptation, stress resistance, and antibiotic resistance (Delgado‐Baquerizo et al. 2021; Shen et al. 2024; Terrat et al. 2017; Liu et al. 2024). Therefore, habitat type, rather than park age or geographical location, appears to be the key driver of divergence in soil microbial functional gene composition. This further corroborates the decisive influence of plant diversity and human management intensity on the functional traits of belowground microbial communities.

Redundancy analysis and variation partitioning further confirmed that habitat type is the most critical factor driving the differences in the composition of the three functional gene categories. Remnant forests, characterized by complex, multi‐layered vegetation structures and higher native species diversity, along with minimal human disturbance, provide diverse ecological niches and stable microenvironments for microbial communities (Foo 2016; Kowarik and von der Lippe 2018; Chen et al. 2021). In contrast, artificial green spaces feature simplified, homogeneous vegetation and undergo frequent, high‐intensity human management (Acar et al. 2007). This aboveground homogenization leads to the standardization of belowground microbial habitats, thereby shaping functional gene profiles dominated by metabolic adaptation and stress resistance (Cen et al. 2025; Li et al. 2023; Liu et al. 2024). Therefore, the highest explanatory power of habitat type for gene composition essentially reflects the strong selective pressure exerted by aboveground vegetation characteristics and human management intensity on belowground microbial functional traits.

Furthermore, the effectiveness of soil nutrients has a significant impact on the composition of functional genes, which can be attributed to the differences in nutrient input and management methods between the two habitat types. In artificial green spaces, long‐term exogenous fertilizer application (such as nitrogen fertilizers) and organic amendments are commonly used to maintain ornamental plant growth, significantly increasing the availability of nutrients like nitrogen and potassium in the soil (Bibi et al. 2019). This eutrophic environment directly selects for and promotes the proliferation of microbial taxa with efficient nutrient metabolism, strong competitiveness, and associations with antibiotic resistance dissemination (Zheng et al. 2025; Pan et al. 2020). In remnant forests, nutrient cycling relies primarily on natural litter decomposition and mineralization—a slower process with stronger homeostasis (Zhou et al. 2025; Zhang et al. 2023). The content and forms of soil total potassium and total nitrogen differ markedly from those in artificial green spaces, thereby sustaining an alternative microbial functional network associated with natural nutrient cycling and plant symbiosis (Liao et al. 2018). Thus, soil total potassium and total nitrogen are not only key soil chemical indicators but also core links connecting aboveground management practices with belowground microbial functional responses. Their content differences profoundly reflect and reinforce the microbial functional gene divergence pattern driven primarily by habitat type.

The results of this study indicate that the widespread enrichment of ARGs in artificial green spaces reflects the continuous selection pressure exerted by intense human management. Such areas typically rely on fertilizers and pesticides to maintain the health of ornamental plants, accompanied by frequent irrigation and soil disturbance (McKeown et al. 2023; Thompson and Kao‐Kniffin 2019). This management regime not only introduces direct exogenous antibiotic selection pressure but also, by altering soil physicochemical properties (e.g., increasing the availability of nutrients like nitrogen and potassium), creates an environment that continuously selects for and enriches antibiotic‐resistant microorganisms and their associated genes (Jacques et al. 2025; Wang et al. 2014; Cen et al. 2025). As the oldest park in this study (established in 1957), Qianlingshan Park has the longest history of management for its artificial green spaces. Consequently, this management‐driven accumulation of ARGs is likely most pronounced here, leading to the significant abundance differences observed between habitats.

In contrast, the enrichment trend of PHIs and VFs in the remaining forests, as well as the particularly prominent differences in habitats in the Huaguoyuan Park, are closely related to the highly complex and stable natural plant communities in remnant forests. These forests possess higher native plant diversity, multi‐layered vegetation structures, and abundant litter, which provide diverse ecological niches and a long‐term co‐evolutionary context for various plant pathogens, symbiotic microbes, and their hosts (Barrico et al. 2018; Vivanco et al. 2018). Within such natural ecosystems, frequent and complex interactions between microorganisms and plant hosts are maintained, thereby sustaining and selecting for a functional gene pool associated with pathogenicity, defense responses, and host adaptation (Cen et al. 2025). The remnant forests in Huaguoyuan Park, potentially due to its specific floristic composition or microenvironmental conditions, may exhibit a more pronounced signal of these interaction‐related genes, thereby amplifying the differences with adjacent artificial green spaces.

The study found that in remnant forests, the abundance of PHIs and VFs was positively correlated with plant species diversity. This relationship stems primarily from the role of complex vegetation systems in shaping microbial interaction networks (Haq et al. 2025; Yu et al. 2026). Remnant forests possess higher native plant diversity and multi‐layered vegetation structures, which not only provide diverse root exudates, litter types, and microhabitats but also create a stable environment for the long‐term co‐evolution of various plant pathogens, endophytes, and their hosts (Barrico et al. 2018; Vivanco et al. 2018). Within such natural communities, higher plant diversity implies more complex biotic interaction interfaces, thereby sustaining and selecting for a functional gene pool associated with host recognition, infection mechanisms, and virulence adaptation. Consequently, the abundance of these two gene categories increases with rising plant diversity.

However, within artificial green spaces, the abundance of all three functional gene categories showed a positive correlation with soil nitrogen content, revealing the strong regulatory effect of anthropogenic nutrient management on microbial functional traits. The vegetation in artificial green spaces is homogeneous and reliant on external inputs for maintenance (Trémeau et al. 2024). Long‐term fertilization and other human interventions significantly elevate soil nitrogen levels. This nitrogen‐rich environment directly selects for microbial taxa with efficient nitrogen metabolic capabilities (Dai et al. 2018). Simultaneously, by altering soil chemical properties and microbial competition dynamics, it collectively promotes the enrichment of multiple functional gene categories, including ARGs, as well as pathogen interaction and VFs (Shen et al. 2024; Terrat et al. 2017). Furthermore, high‐nitrogen environments are often accompanied by the input of organic fertilizers. The integrons and mobile genetic elements carried by these fertilizers can facilitate horizontal gene transfer of ARGs, PHIs, and VFs. At the same time, nitrogen metabolites can exert co‐selection pressure on resistant strains, thereby causing these genes to accumulate in nitrogen‐rich soils (Zhu et al. 2017; Heydari et al. 2022). This indicates that in human‐managed, simplified ecosystems, soil chemical factors—such as nitrogen content—act as key environmental filters driving the composition of microbial functional genes.

Building upon the foundational work of Cen et al. (2025), which revealed the impact of habitat type conversion on overall soil microbial functional gene diversity, this study represents a significant step forward in depth and focus. We specifically targeted three core functional gene categories with clear ecological and public health implications, thereby elevating the research from ecological observation to health diagnostics. This study not only quantitatively confirmed that habitat type is the primary driver shaping the composition of these functional gene categories, but also revealed distinct underlying mechanisms through correlation analysis. In remnant forests, gene abundance was positively correlated with plant diversity, underscoring the central role of natural biotic interactions. In artificial green spaces, gene abundance was positively correlated with soil nitrogen content, highlighting the selective pressure of anthropogenic nutrient management. This shift from overall functional diversity to key ecologically relevant gene groups advances the study of habitat conversion effects to a level with clearer implications for health risk assessment and ecological process indication. It should be noted that the presence of genes does not equate to an active risk of pathogens, but the enrichment of these functional genes can serve as an early warning indicator for potential health risks. Nevertheless, this study still provides direct and relevant specific scientific evidence for the functional health of soil microorganisms, thereby contributing to the sustainable management of karst urban parks.

Based on our findings, we propose targeted management strategies for urban karst mountain parks to mitigate potential ecological and health risks. For artificial green spaces, management should focus on reducing exogenous nitrogen input by optimizing fertilization practices. This is crucial to lower soil nitrogen levels, thereby suppressing the enrichment of ARGs and other pathogenicity‐related genes linked to nutrient‐driven microbial selection. For remnant forests, the primary goal is to preserve their high native plant diversity and complex structure, which underpin the unique soil microbiome and its functions. To address the elevated abundance of pathogen–host interaction genes in these areas, practical measures such as installing educational signage to discourage direct soil contact and promoting post‐visit hand hygiene among park users are recommended to minimize potential exposure.

The present study is subject to several limitations. Firstly, the analysis was primarily based on gene composition and was not validated through microbial activity or gene expression levels. Secondly, the study encompassed a single season, thus disregarding the potential for temporal variations. Finally, detailed management records, particularly nitrogen fertilizer application rates and frequencies, were not available for the artificial green spaces. Therefore, we were unable to propose quantitative management recommendations, such as a specific percentage or absolute reduction target for nitrogen input. Future research should incorporate metatranscriptomic or proteomic methods to verify the expression of functional genes and the actual activity of microorganisms. Concurrently, multi‐seasonal sampling should be conducted to capture the temporal dynamics of soil microbial communities. In addition, subsequent studies may concentrate on the exact management of nitrogen inputs by analyzing the associations between particular microbial groups and their functions, and the age of the parks, as well as their maintenance practices. This would provide quantitative recommendations for the management of urban green spaces.

## 5 Conclusion

This study investigated three categories of ecologically and health‐relevant functional genes in soil microorganisms within karst mountain parks, providing critical evidence for assessing soil microbial health risks in these urban environments. To date, metagenomic studies linking habitat transformation to health‐relevant microbial genes in karst urban parks remain scarce, and this study helps fill that important knowledge gap. Our findings demonstrate that the distribution of these key functional genes in karst mountainous urban parks is primarily governed by habitat type. Specifically, remnant forests, characterized by high plant diversity, are enriched with genes related to PHIs and VFs. Conversely, in artificial green spaces, anthropogenic management practices that enrich soil nitrogen significantly promote the accumulation of ARGs. To address the associated ecological and health risks, management strategies should aim to reduce exogenous nitrogen inputs in artificial green spaces and enhance public health protection awareness in remnant forest areas. Future research should not only detect the presence of genes but also measure gene expression or microbial activity and extend investigations to diverse ecosystems to validate the universality of these findings.

## Author Contributions

**Weize Wang:** data curation (equal), investigation (equal), methodology (equal), software (equal), validation (equal), visualization (equal), writing – original draft (equal), writing – review and editing (equal). **Chunhua Cen:** data curation (equal), investigation (equal), methodology (equal), writing – review and editing (equal). **Jingyi Yang:** conceptualization (equal), data curation (equal), funding acquisition (equal), methodology (equal), project administration (equal), resources (equal), supervision (equal), validation (equal), writing – review and editing (equal).

## Ethics Statement

The authors have nothing to report.

## Conflicts of Interest

The authors declare no conflicts of interest.

## Supporting information


**Table S1:** Sample sequence coverage.

## Data Availability

The data for this study are available via the Mendeley Data Repository, https://doi.org/10.17632/pnf2s9r7t6.1.
