# Supplementary material for: Dominant Role of Habitat Transformation in Driving the Divergence of Health‐Risk Related Microbial Functional Genes in Karst Mountain Parks: A Metagenomic Study
Source: Ecol Evol. 2026 Jul 29;16(8):e74112. doi: 10.1002/ece3.74112 (PMC13416750; doi:10.1002/ece3.74112)
Supplement: Supplementary file 1 — Table S1: Sample sequence coverage. [file ECE3-16-e74112-s001.docx]

**Supplementary material**

Table S1 Sample sequence coverage

| sample | goods_Coverage | sample | goods_Coverage | sample | goods_Coverage |
| --- | --- | --- | --- | --- | --- |
| QLS1-1 | 0.999996187 | HGY1-1 | 0.999970727 | YLH1-1 | 0.999990509 |
| QLS1-2 | 0.999993603 | HGY1-2 | 0.999943265 | YLH1-2 | 0.999992244 |
| QLS1-3 | 0.999985239 | HGY1-3 | 0.99998302 | YLH1-3 | 0.999977807 |
| QLS1-4 | 0.999977909 | HGY1-4 | 0.999974131 | YLH1-4 | 0.999956007 |
| QLS1-5 | 0.999977899 | HGY1-5 | 0.999984836 | YLH1-5 | 0.999978895 |
| QLS1-6 | 0.999979086 | HGY1-6 | 0.999969285 | YLH1-6 | 0.999978524 |
| QLS1-7 | 0.999981685 | HGY1-7 | 0.999982992 | YLH1-7 | 0.9999655 |
| QLS1-8 | 0.999978153 | HGY1-8 | 0.99998275 | YLH1-8 | 0.999980374 |
| QLS1-9 | 0.999981217 | HGY1-9 | 0.999983225 | YLH1-9 | 0.999996069 |
| QLS1-10 | 0.999984669 | HGY1-10 | 0.999974008 | YLH1-10 | 0.999982112 |
| QLS2-1 | 0.9999577 | HGY2-1 | 0.999985495 | YLH2-1 | 0.99996832 |
| QLS2-2 | 0.999979612 | HGY2-2 | 0.999968767 | YLH2-2 | 0.99998153 |
| QLS2-3 | 0.99998312 | HGY2-3 | 0.999954261 | YLH2-3 | 0.999982273 |
| QLS2-4 | 0.999978376 | HGY2-4 | 0.999976595 | YLH2-4 | 0.999979735 |
| QLS2-5 | 0.999976301 | HGY2-5 | 0.999948169 | YLH2-5 | 0.999983456 |
| QLS2-6 | 0.999966109 | HGY2-6 | 0.999979836 | YLH2-6 | 0.999963749 |
| QLS2-7 | 0.999962558 | HGY2-7 | 0.999963011 | YLH2-7 | 0.999989399 |
| QLS2-8 | 0.999969854 | HGY2-8 | 0.999976424 | YLH2-8 | 0.999981215 |
| QLS2-9 | 0.999987849 | HGY2-9 | 0.999972057 | YLH2-9 | 0.999956336 |
| QLS2-10 | 0.999975811 | HGY2-10 | 0.999959044 | YLH2-10 | 0.99998353 |

Notes: The “goods_coverage” is an indicator reflecting the sequencing depth. The closer its value is to 1, the more reasonable the sequencing depth is, meaning that this depth has basically covered all the species in the sample.
